# Potassium Ethylate

**Published:** 1894-09-01

**Authors:** Benjamin Ward Richardson


### Potassium Ethylate.

By Sir Benjamin Ward Richardson, M.D., F.R.S.

Sodium ethylate has been under review in these pages, and up to the present time it alone has made its way in medicine. Potassium ethylate, although described truthfully by me so long as twenty-five years ago, has, I believe, never been used by any one else. I am bound to say of it that it is a remarkable preparation, and is destined to play an important part when its uses are better known. It is made precisely in the same way as the sodium salt, with the single difference that potassium takes the place of sodium. It will crystallize like the sodium salt, but it is quite efficient when employed in the state of solution. It can be applied in the same manner, either with a glass rod, a brush, or a pointed quill, as the sodium ethylate solution, and the same rule must be carefully followed in regard to the avoidance of water on the parts acted upon by it.

The difference in action between the two salts is another good illustration of the effect of difference of constitution. Potassium plays a different part to sodium, and its ethylate therefore differs in its effect. Potassium is a keener caustic, and the ethylate shares in the self-same property. On the skin the potassium ethylate cuts almost like a knife, destroys almost like the actual cautery, it is so incisive in its power. Exposed to a moist surface, the potassium seizes the water so rapidly that the caustic potassa *in nascente* acts as a caustic in an instant, the liberated alcohol coagulating the destroyed tissue as rapidly. The result is that in cases where the sodium ethylate is not sufficiently effective the potassium takes its place in a more determinate manner.

A young lady was brought to me who had on the inside of one thigh a hard growth or excrescence, which measured in circumference the size of a full-grown orange. If it be supposed that an orange divided in half had been passed under the skin with the convex or rind surface uppermost, a good idea is given of the shape and dimensions of the growth. The growth was darkish brown in colour, and was almost horny in its hardness. It had appeared at birth, of small size, and was considered to be a large wart. As the limb increased in size the growth enlarged with it, until at twenty years it had become of the character described above. It gave no actual pain, but it was felt by the patient to be most objectionable, and she desired its removal. An eminent surgical friend whom she consulted did not advocate its removal by the knife, but thought it could be removed like a nævus by the sodium ethylate, and he suggested that I should undertake the trial I did so, but with little effect, for the structure of the mass was so condensed that there was feeble action, and, in fact, a difficulty in making an abrated surface. After several non-effective attempts with the sodium salt I moved to the potassium, and with distinct success. After the first free application a crust was formed; that was removed, and without pain a second encrustation was secured, then a third, and so on, until a sensitive surface was reached. At this stage the potassium seemed to be too active; there was some surface bleeding and too rapid a destruction. The sodium ethylate was applied, and under it a complete and satisfactory cure was carried out. In this case the applications of the potasium ethylate were made at intervals of three days for seven weeks, and in the end the edges of natural skin gradually contracted and closed in so well that in a few months it was difficult to suppose there ever had been so large a growth, so limited and smooth a cocatrix followed.

The observation made about loss of blood after the application of the potassium ethylate leads to a special word on the subject. The potassium ethylate destroys so rapidly there is some risk of exudation of blood. From some observations on the effect of it upon segments of the aorta of the ox I do not think it is capable of dividing the arterial tunics but on vascular growths filled with small vessels it has a destructive action which requires to be remembered. The late Mr. Lord, of Hampstead, asked me to destroy three pointed vascular growths or tumours that had formed on the head of one of his patients, a woman about sixty years of age. The growths were not cystic, but each one had a vascular surface, a base the size of a shilling, an elevation of nearly half-an-inch, and a rather pointed apex. They partook of the character in each case of a large vascular nævus, increasing rapidly in size. In order to destroy it rapidly I treated one growth with potassium ethylate, with the effect of causing quickly a large and deep crust which was left in the ordinary way; but in the course of an hour I was sent for because of bleeding. Although the patient had sat quietly, the crust that was formed by the caustic had separated and was raised two or three lines from a bleeding surface. Half-an-ounce of blood at least had been lost. I removed the crust and stopped the oozing of blood at once by a spray of ether and tannin to which a little alcohol had been added, and a few days later on removing a dressing of cotton-wool and tannin that had been finally laid on the bleeding surface, I was agreeably surprised to find there was a practical cure, nothing remaining except a slightly raised vascular surface which was easily removed by the sodium ethylate. The other tumours were removed by the sodium salt alone with complete success.

The ethylates can be used with good effect for the treatment of polypus growths of the nasal cavity if the growth is easily within reach. The plan consists in making a small brush of cotton, by tessing the cotton out of the end of a small wax or paraffin taper. The brush, so formed, is dipped into the ethylate solution and (using the taper as if it were a bougie), plunging the end of the brush right into the polypus and holding it there a minute or two. If the polypus be not extremely large it may, in this manner, be destroyed immediately, and may come away as an encrustation, a result which occurred in the case of a young gentleman who was brought to me by my friend Dr. James Stevenson. It is not always, however, we are so fortunate; as a general rule it is necessary to renew the application two or three times before complete removal is effected. The sodium ethylate acts best for this operation.

I leave these ethylates once again for practice. They stand firmly on a physical method, a method almost as mechanical as that of the knife itself. They admit of being used locally for cancer as well as for nævus. They are powerfully antiseptic, and in their direct action on blood they dissolve the red corpuscles vigorously, but the white corpuscles with comparative slowness.

